# Natural Plant Compounds: Does Caffeine, Dipotassium Glycyrrhizinate, Curcumin, and Euphol Play Roles as Antitumoral Compounds in Glioblastoma Cell Lines?

**DOI:** 10.3389/fneur.2021.784330

**Published:** 2022-02-17

**Authors:** Gabriel Alves Bonafé, Matheus Negri Boschiero, André Rodrigues Sodré, Jussara Vaz Ziegler, Thalita Rocha, Manoela Marques Ortega

**Affiliations:** ^1^Laboratory of Cell and Molecular Tumor Biology and Bioactive Compounds, São Francisco University Medical School, São Paulo, Brazil; ^2^Verdi Cosmetics LLC, São Paulo, Brazil; ^3^Postgraduate Program in Biomaterials and Regenerative Medicine, Faculty of Medical Sciences and Health, Pontifical Catholic University of São Paulo, São Paulo, Brazil

**Keywords:** caffeine, dipotassium glycyrrhizinate, curcumin, euphol, microRNAs, glioblastoma cell lines

## Abstract

Many plant-derived compounds are shown to be promising antitumor therapeutic agents by enhancing apoptosis-related pathways and cell cycle impairment in tumor cells, including glioblastoma (GBM) cell lines. We aimed to review four natural plant compounds effective in GBM cell lines as caffeine, dipotassium glycyrrhizinate (DPG), curcumin, and euphol. Furthermore, antitumoral effect of these plant compounds on GBM cell lines through microRNAs (miRs) modulation was investigated. However, only DPG and curcumin were found as effective on miR modulation. Caffeine arrests GBM cell cycle in G0/G1 phase by cyclin-dependent kinases (CDK) complex inhibition and by decreasing *BCL-2* and increasing *FOXO1* expression levels causing greater apoptotic activity. Caffeine can also directly inhibit IP3R3, p38 phosphorylation, and rho-associated protein kinase (ROCK), decreasing cell invasion and migration capacity or indirectly by inhibiting the tissue inhibitor metalloproteinase-1 (*TIMP-1*) and integrins β1 and β3, leading to lower matrix metalloproteinases, MMP-2 and MMP-9. DPG presents antitumoral effect in GBM cells related to nuclear factor kappa B (NF-κB) pathway suppression by *IRAK2* and *TRAF6*-mediating miR-16 and miR-146a, respectively. More recently, it was observed that DPG upregulated miR-4443 and miR-3620, responsible for post-transcriptional inhibition of the NF-κB pathway by *CD209* and *TNC* modulation, respectively leading to lower MMP-9 and migration capacity. Curcumin is able to increase miR-223-3p, miR-133a-3p, miR-181a-5p, miR-34a-5p, miR-30c-5p, and miR-1290 expression leading to serine or threonine kinase (AKT) pathway impairment and also it decreases miR-27a-5p, miR-221-3p, miR-21-5p, miR-125b-5p, and miR-151-3p expression causing p53-BCL2 pathway inhibition and consequently, cellular apoptosis. Interestingly, lower expression of miR-27a by curcumin action enhanced the C/EBP homologous protein(CHOP) expression, leading to paraptosis. Curcumin can inhibit miR-21 expression and consequently activate apoptosis through caspase 3 and death receptor (DR) 4 and 5 activation. Autophagy is controlled by the LC-3 protein that interacts with Atg family for the LC3-II formation and autophagy activation. Euphol can enhance LC3-II levels directly in GBM cells or inhibits tumor invasion and migration through PDK1 modulation.

## Introduction

The central nervous system (CNS) tumors account for about 3% of all neoplasms (1). Glial tumors or gliomas comprise 50% of all CNS tumors and 80% of all brain-initiating malignant tumors (2). Gliomas are subdivided into astrocytomas, oligodendrogliomas, and ependymomas (3). Furthermore, according to the World Health Organization (WHO), astrocytomas are divided into four degrees of malignancy, which are based on histopathological criteria such as the presence of atypical cells, mitosis, endothelial proliferation, and necrosis; and molecular depending on the presence or absence of mutations in the isocitrate dehydrogenase 1 and 2 genes (*IDH1* and *IDH2*) (4).

Malignant gliomas are the most common primary brain tumor, representing 42% of CNS tumors (5). The most common aggressive glioma form is known as glioblastoma (GBM) or WHO grade IV has a 12–15-month medium survival rate. The lower-grade gliomas (WHO grades II and III) appear less aggressive at the time of diagnosis but eventually progress into a malignant phase within 5–10 years (6). Despite the surgical procedures and treatment regimens with radiation and chemotherapy, malignant gliomas remain incurable (6), due to their resistance to all conventional therapies and the diffuse infiltrative nature of the tumor cells.

Therefore, new therapies and therapeutic combinations need to be developed and quickly approved for use in patients. However, to gain approval, therapies need to be safe, effective, and able to penetrate the blood–brain barrier (BBB) (7). In that way, natural treatments might be new compounds that can eliminate GBM development and tumor expansion.

Many plant-derived compounds are shown to be promising antitumor therapeutic agents by enhancing apoptosis-related pathways in tumor cells through the regulation of microRNAs (miRs) expression (8, 9).

MicroRNAs are small molecules (on average about 22 nucleotides) of non-coding RNA that bind to complementary sequences in the 3'UTR portion of the transcribed mRNA target resulting in translational repression or gene degradation and silencing (10). The human species is able to synthetized ~2,600 mature miRs (11), and more than 50% of these miRs are located in cancer-associated genomic regions (12).

The deregulation of miRs is associated with the development and progression of several types of tumors (13), including GBM (14), by inhibiting the translation of its target genes (10). Recent evidence suggests that miRs play an essential role in the etiology of GBM, as they are involved in several biological processes, such as cell growth, migration and invasion, apoptosis, and cell differentiation (15).

Therefore, in this study, we aimed to review four natural plant compounds that may be effective in enhancing apoptosis-related pathways and cell cycle impairment in GBM cell lines. Furthermore, antitumoral effect of these plant compounds on GBM cell lines through miR modulation was investigated, although only DPG and curcumin were found as effective on miR modulation.

## Methods

Following the recommendations from Preferred Reporting Items for Systematic Review and Meta-Analysis (PRISMA), a systematic review of the studies published in the PubMed (http://www.ncbi.nlm.nih.gov/pubmed) database in the last 10 years was conducted. Experimental studies that included natural plant compounds as caffeine, dipotassium glycyrrhizinate (DPG), curcumin, and euphol in gliomas were included (19 studies) (Table 1). In addition, studies related to these natural compounds and miR effects were also selected for the present review (7 studies) (Table 2).

**Table 1:** The effects of the natural compounds on GBM cell lines (19–22, 41, 47, 49, 54, 63, 69, 83–87, 89, 92, 101).

**Natural compounds**	**Cell lines**	**Studies**	**Assays**	**Effects**	**Pathways**
**Caffeine**					
	C6/U87MG	Jiang et al. (16); Liu et al. (17)	*in vitro*	Cell cycle arrest; proliferation inhibition; apoptosis stimulation	G0/G1 phase arrest; S phase decreased; ↓Blc-2; ↑CytC e caspase 3
	U87MG/U178MG/ T98G/U373MG/M059K	Kang et al. (18)	*in vitro*/ *in vivo*	Migration and invasion inhibition	↓IP3R3-mediated Ca2+ release
	U87MG	Ku et al. (19)	*in vitro*	Cell cycle arrest; proliferation inhibition; apoptosis stimulation	G0/G1 phase arrest; ↓Rb phosphorylation; ↑caspase 3 and PARP activation; ↑GSK3β phosphorylation
	U87MG/GBM8401 /LN229	Cheng et al. (20)	*in vitro*	Migration and invasion inhibition	↑TIMP-1; ↓MMP-2; ↓p-ERK and integrins β1 and β3
	U251	Sun et al. (21)	*in vitro*	Apoptosis stimulation; proliferation inhibition	↑FoxO1 expression; ↑proapoptotic target Bim
**AC derivatives**					
PT93	T98G/U87MG /U251/HT22	Li et al. (22)	*in vitro*	Apoptosis stimulation; proliferation inhibition; migration inhibition	↓extracellular MMP-2 and MMP-9
FLVM/FLVZ	U87MG	Khan et al. (23)	*in vivo*	Apoptosis stimulation; proliferation inhibition; angiogenesis inhibition	↓tumor hypoxia (HIF-1α); ↓angiogenesis (CD34, VEGF, IL17A); ↓cell proliferation (Ki67); ↑Bax, caspase and FasL
GA					
	U251	Li et al. (24)	*in vitro*	Apoptosis stimulation; survival rate and colony formation inhibition;	↓NF-κB-p65 reduction
DPG					
	U87MG/T98G	Bonafé et al. (25)	*in vitro*	Apoptosis stimulation; proliferation inhibition; steam cells formation inhibition	↓NF-κB by *IRAK-2* and *TRAF6* reduction
	U251//U138MG	Unpublish data	*in vitro*	Apoptosis stimulation; proliferation inhibition; steam cells formation inhibition, migration inhibition	↓NF-κB by *CD209* and *TNC* modulation
Curcumin					
	U87MG	Wu et al. (26)	*in vitro*	Proliferation inhibition; apoptosis stimulation	↓NF-κB suppression
	U87MG	Li et al. (27)	*in vitro*/*in vivo*	Proliferation inhibition; apoptosis stimulation	↓MAPK pathway by phosphorylation of p38; ↑Bax and cytochrome C; ↓PCNA as reduction of cell proliferation
	U87MG/U251	Yin et al. (28)	*in vitro*/ *in vivo*	Proliferation inhibition; apoptosis stimulation; migration inhibition	SHH/GLI1 pathway has also been shown to regulate the stemness and invasiveness
	C6	Tan et al. (29)	*in vitro*/ *in vivo*	Apoptosis stimulation; tumor growth inhibition	↓*PDCD4* and *PTEN* inhibition
	GSC	Qian et al. (30)	*in vitro*/ *in vivo*	Apoptosis stimulation; cell growth inhibition	↑miR-145 results in increased cell growth inhibition and apoptosis to DC
	A172	Garrido-Armas et al. (31)	*in vitro*	Paraptosis	↑several miRs downregulate the AKT and p53-BCL2 pathways; ↓miR-27a expression reduced CHOP leading the cells to paraptosis
Euphol and its derivatives					
		Silva et al. (32)	*in vitro*/ *in vivo*	Proliferation and cell motility inhibition	↑autophagy-associated protein LC3-II and acidic vesicular organelle formation with Bafilomycin A1 potentiating cytotoxicity
IngA/IngB/IngC	12 human gliomas and GBM^*^	Silva et al. (33)	*in vitro*	Proliferation inhibition	Dose-dependent cytotoxic effects

Cyt C, cytochrome C; AC, caffeic acid derivative; GA, glycyrrhizic acid; DPG, dipotassium glycyrrhizinate; DC, demethoxycurcumin; ^*^, U87MG, U373, U251, GAMG, SW1783, SNB19; Cdk, cyclin-dependent kinases; ROCK, rho-associated protein kinase; p-p38, phosphorylated p38; TIMP-1, tissue inhibitor metalloproteinase-1; β, Integrins; MMP, matrix metalloproteinase; IngA, ingenol-3-transcinnamate; IngB, ingenol-3-hexanoate; IngC, ingenol-3-dodecanoate. The meaning of the symbols ↑, ↓ are up-regulated and down-regulated respectively.

**Table 2:** The natural compound effects on miRs in GBM cell lines (83–87, 89).

**Natural compounds**	**Cell lines**	**Studies**	**Assays**	**miR effects**	**Pathways**
**DPG**					
	U87MG/T98G	Bonafé et al. (25)	*in vitro*	↑miR-146a ↑miR-16	↓NF-κB by upregulating miR16 and miR146a, which downregulate its target genes, *IRAK2* and *TRAF6*
	U251/U138MG	Unpublished data	*in vitro*	↑miR-4443 ↑miR-3620	↑ miR-4443 and ↑miR-3620 induces post-transcriptional inhibition of the NF-κB by *CD209* and *TNC* genes modulation and leading to an antimigratory effect on GBM cells
**Curcumin**					
	U87MG	Wu et al. (26)	*in vitro*	↑miR-146a	↑miR-146a enhances apoptosis and suppressed NF-κB activation
	U87MG	Li et al. (27)	*in vitro*/ *in vivo*	↑miR-378	↑miR-378 enhances the response to curcumin by targeting *P-P38*
	A172	Garrido-Armas et al. (31)	*in vitro*	↑miR-223-3p ↑miR-1290 ↑miR-34a-5p ↑miR-181a-5p ↑miR-133a-3p ↑miR-30c-5p ↓miR-27a-3p ↓miR151-3p ↓miR-221-3p ↓miR-21-5p ↓miR-125b-5P	↑miRs downregulate the AKT and p53-BCL2 pathways; ↓miR-27a expression reduced CHOP leading the cells to paraptosis
	U87MG/U251	Yin et al. (28)	*in vitro*/ *in vivo*	↑miR-326	↑miR-326 enhances curcumin-inhibition by SHH/GLI1 and regulated the expression of p53 and stemness; tumor reduction
	C6	Tan et al. (29)	*in vivo*/ *in vitro*	↓miR-21	PDCD4 and PTEN were induced in the miR21ASO/DP and miR21ASO/DP-curcumin complex
	GSC	Qian et al. (30)	*in vitro*/ *in vivo*	↑miR-145	↑miR-145 is involved in enhancing chemosensitivity to miR-145 by targeting the SOX2-Wnt/β-catenin axis

*miR21ASO/DP, miR-21 antisense oligonucleotide. The meaning of the symbols ↑, ↓ are up-regulated and down-regulated respectively*.

## Discussion

### Does Caffeine Play Role on GBMs?

**Figure 1:**
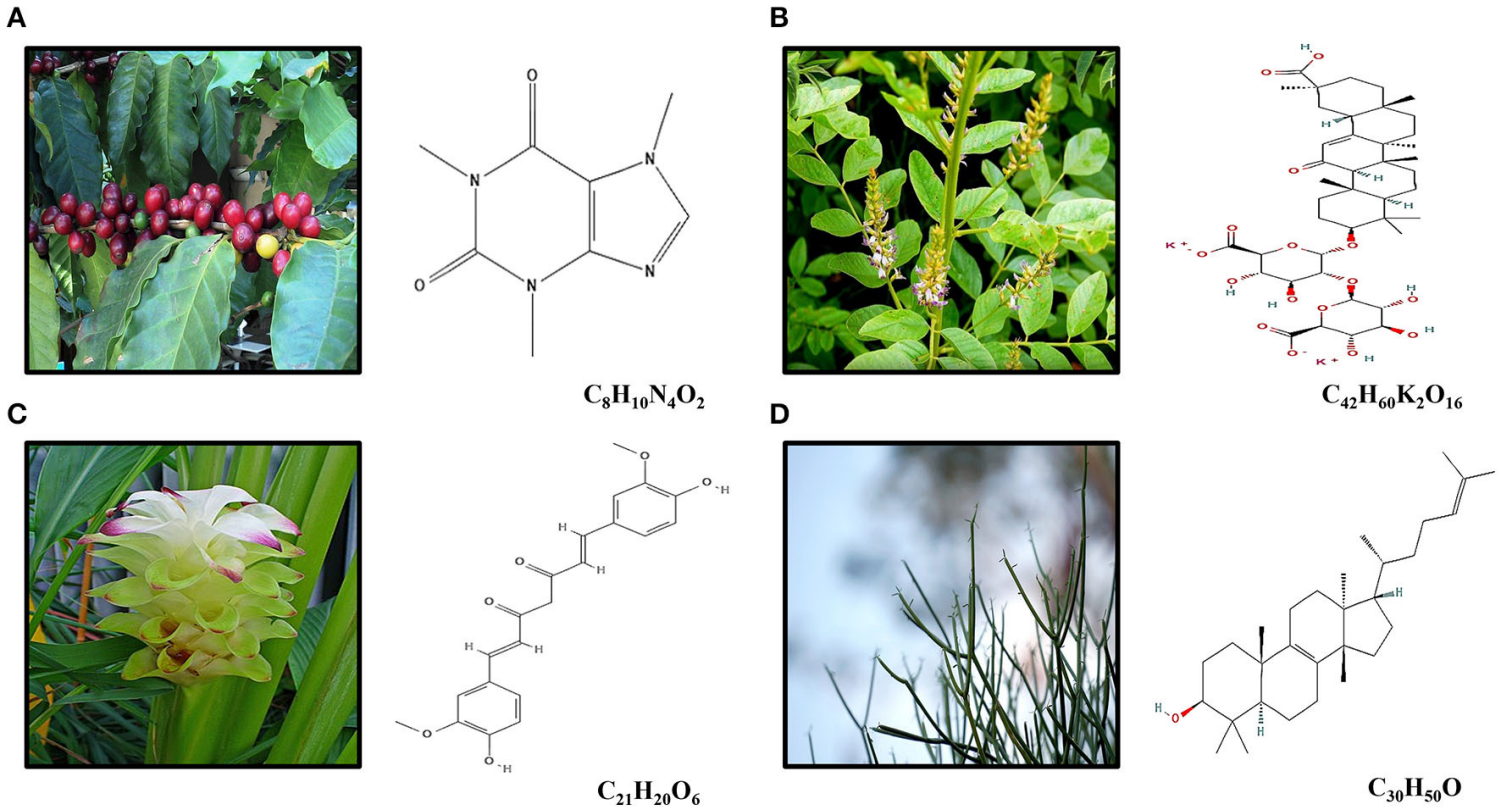
**(A)** Caffeine (C_8_H_10_N_4_O_2_) is an alkaloid occurring naturally in some 60 plant species, of which cocoa beans, kola nuts, tealeaves, yerba maté, guarana berries, guayusa, yaupon holly, and coffee beans are the most well-known. The best-known source of caffeine is the coffee bean, the seed of the coffee plant. **(B)** Licorice root (*Glycyrhiza glabra*) is widely used in traditional Chinese medicine for its pharmacological and physiological action as antiallergic, antibiotic, antiinflammatory, and antitumor effects. DPG (C_42_H_60_K_2_O_16_) is a dipotassium salt of GA, a compound isolated from licorice root. **(C)** Curcumin (diferuloylmethane; C_21_H_20_O_6_) is the main compound found in turmeric, an Indian spice derived from *Curcuma longa Linn*, which presents antioxidant and antiinflammatory effects. **(D)** The euphol (C_30_H_50_O), a tetracyclic triterpene alcohol, is the main constituent of the *Euphorbia tirucalli* known as aveloz.

Caffeine (C_8_H_10_N_4_O_2_) is a methylxanthine compound commonly found in coffee and tea (Figure 1A), which are the most ingested neuroactive substance in the world (34). Caffeine possesses antiinflammatory and antioxidant effect, as acetylcholinesterase inhibitors (35). Recently, it was reported that caffeine consumption might be associated with lower glioma risk (36). In fact, caffeine has been associated with rat glial cell (C6) and human GBM cell lines (U251 and U87MG) growth inhibition and apoptosis activation (16, 17, 19, 21).

#### Effects of Caffeine in Cell Cycle

**Figure 2:**
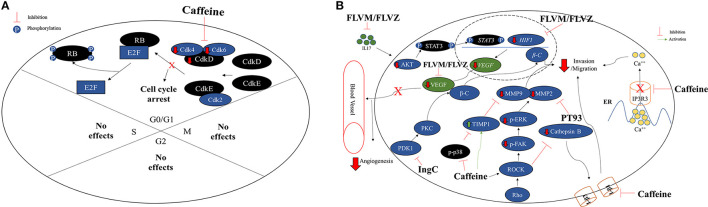
**(A)** Caffeine arrests the cell cycle on G0/G1 phase in GBM cell lines. Normally, GO/G1 cell cycle phase is regulated by the CdkD and CdkE, which interacts with Cdk4, Cdk6 and Cdk2 forming CdkD/Cdk4,6 and CdkE/Cdk2 complexes. The CDK complexes are responsible for retinoblastoma (Rb) inactivation through phosphorylation and consequently, releasing the transcriptional factor E2F, retaining the cell cycle active. Caffeine appears to arrest the cell cycle in G0/G1 phase in GBM cell lines C6, U251, and U87MG by CDK complex inhibition. No caffeine effects were observed on S, G2, or M phases. **(B)** Effects of caffeine and compounds derived from caffeine on invasion/migration capacity and angiogenesis impairing in GBM cell lines. The caffeine can inhibit the IP3R3, an ER calcium receptor, which leads to a lower concentration of intracellular calcium, decreasing cell invasion and migration capacity. The rho-associated protein kinase (ROCK) cathepsin B/FAK/ERK pathway, associate with cellular invasion and migration, is also inhibited by caffeine through targeting phosphorylated p38 (p-p38) and ROCK proteins direct or by indirect inhibition through the TIMP-1 and integrins β1 (I-β1) and I-β3, leading to lower MMP-2 and MMP-9. In addition, PT93, a compound derived from CA, acts direct in the inhibition of MMP-2 and contributing to lower cellular invasion or migration capacity. In the same way, the CA derivatives FLVM/FLVZ can either inhibit IL17 and HIF-1, leading to VEGF inhibition and angiogenesis impairing. The euphol derivative (IngC), on the other hand, acts in PKC and Wnt/β-catenin (β-C) pathways, inhibiting the PDK1, leading to lower concentration of β-C, an effector protein, consequently inhibiting the tumor invasion and migration.

The cell cycle checkpoints are controlled by cyclin and cyclin-dependent kinases (CDK) proteins. Generally, during final G1 phase, the cyclins D and E are synthetized, which associates with Cdk4 and/or Cdk6 and Cdk2 cyclins, respectively, forming CdkD/Cdk4,6 and CdkE/Cdk2 complexes (37). Both complexes participate in the phosphorylation and inactivation of the retinoblastoma protein (Rb). After phosphorylation, Rb unbound the transcription factor E2F protein, responsible for cyclin A and cyclin E synthesis (38) (Figure 2A). Caffeine effect on cell cycle might be associated with the suppression of CdkD/Cdk4,6 complex and subsequently phosphorylation (p) and inactivation of retinoblastoma (Rb) (39), preventing the E2F transcription factor unbound and the synthesis inhibition of important cell cycle proteins (40). In fact, it was observed that C6 (0.5 mM) and U87MG (1–5 mM) GBM cell lines presented lower S phase and G0/G1 phase arrested in a caffeine dose-dependent way (16, 17, 19, 21) (Figure 2A). Kaufmann et al. (41) have confirmed that caffeine actually targets the CdkD/Cdk4,6 complex required -for inactivation of pRB, in telomerase-expressing human fibroblasts.

In addition, the glycogen synthase kinase-3beta (GSK-3β) substrate phosphorylates the cyclin D1, leading to its degradation, decreasing Cdk4 activity, which leads to the S phase (19, 42). Hashimoto et al. (39) have suggested that the caffeine inhibitory effect on cell growth was mediated through GSK-3β by direct inhibition of PI3-kinase upstream. Interestingly, it was reported that caffeine, in combination with temozolomide (TMZ), revealed synergistic effects in U87MG cell line, since the combinational therapy TMZ suppressed the phosphorylation of ATM and p53 and downregulated p21 expression, thus releasing DNA-damaged cells from G2 arrest into premature mitosis. Cell cycle analysis demonstrated that the proportion of cells arrested in G2 phase decreased when caffeine was administered together with TMZ. In conclusion, the authors have demonstrated that caffeine enhanced the efficacy of TMZ through mitotic cell death by impeding ATM/p53/p21-mediated G2 arrest (43). The effects of caffeine on GBM cell cycle regulation are summarized in Figure 2A.

#### Effects of Caffeine in Apoptosis

It has been reported that caffeine has an anticancer apoptotic effect in various types of cancer such as gastric cancer (17), neuroblastoma (44), and GBM (16, 17, 19, 21).

**Figure 3:**
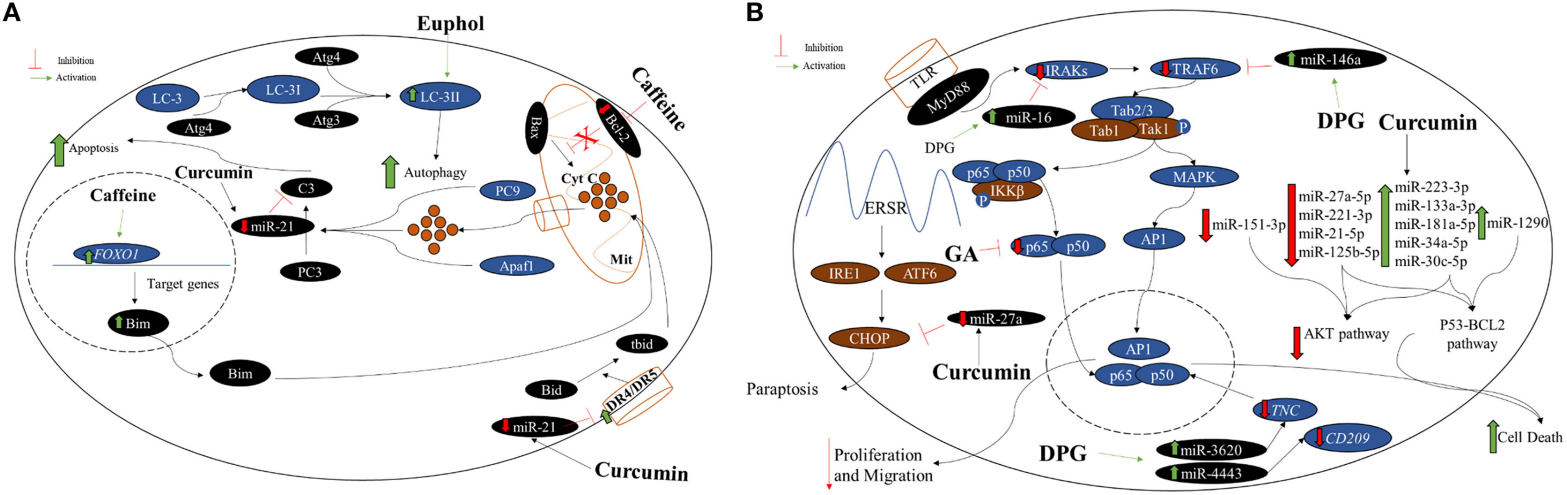
**(A)** Caffeine, curcumin, and euphol antitumoral effect on GBM cell lines. Generally, the mitochondrial apoptotic pathway is regulated by the proapoptotic protein Bax, which acts in the dissociation of cytochrome C (Cyt C) in the mitochondria (Mit). Thus, Cyt C along the procaspase 9 (PC9) and Apaf1, activate procaspase 3 (PC3) into caspase 3 (C3) leading the cell to apoptosis. Moreover, the nuclear protein Foxo1 acts in the transcription of Bim, which contributes to the dissociation of Cyt C and apoptosis stimulation. Therefore, the ratio Bax/Bcl-2 is an excellent indicative of cellular apoptosis. Caffeine acts decreasing *BCL-2* and increasing *FOXO1* expression levels causing greater apoptotic activity. Curcumin can inhibit miR-21 expression and consequently activating apoptosis by caspase 3 and death receptor (DR) 4 and 5 activations. Autophagy, a misfolded proteins degradation and elimination and also damaged organelles, is control by the LC-3 protein that interacts with Atg family for the LC3-II formation and autophagy activation. Euphol is able to enhance LC3-II levels direct in GBM cell lines. **(B)** Effects of GA, DPG, and curcumin on apoptosis, migration, and paraptosis in GBM cell lines. GA, a compound isolated from licorice root, has presented inhibition effect on cell proliferation and colony formation in U251 GBM cell line. In addition, GA also presented apoptosis stimulation in these cells. The GA antitumoral effect is by direct p65 protein inhibition, responsible for nuclear factor kappa B (NF-κB) pathway activating. More recently, DPG, a dipotassium salt of GA, also has presented antitumoral effect related to NF-κB pathway suppression by *IRAK2* and *TRAF6*-mediating miR-16 and miR-146a, respectively, in U87MG and T98G cells. More recently, it was observed that DPG upregulated miR-4443 and miR-3620, responsible for post-transcriptional inhibition of the NF-κB pathway by *CD209* and *TNC* modulation, respectively, in U251 and U138MG, leading to lower MMP-9. The curcumin effect was able to increase miR-223-3p, miR-133a-3p, miR-181a-5p, miR-34a-5p, miR-30c-5p, and miR-1290 expression leading to serine or threonine kinase pathway (AKT) pathway impairment. Curcumin effect also decreased miR-27a-5p, miR-221-3p, miR-21-5p, miR-125b-5p, and miR-151-3p expression causing p53-BCL2 pathway inhibition and consequently, GBM cell death. Interestingly, lower expression of miR-27a by curcumin action enhanced the CHOP protein expression, leading to paraptosis.

The balance between the proapoptotic proteins, Bax, Bak, Bim, and Bad, and antiapoptotic proteins, Bcl-2 and Bcl-xL, regulates the apoptotic pathway (45). These proapoptotic and antiapoptotic proteins are responsible for cytochrome C (Cyt C) dissociation from the external mitochondrial membrane and prevent the Cyt C dissociation, respectively (46, 47). Therefore, the ratio Bax/Bcl-2 is an excellent indicative of apoptosis in tumor cells (46, 47) (Figure 3A).

Two studies have showed a decreased expression of antiapoptotic Bcl-2 and no changes on proapoptotic protein Bax, indicating an imbalance of Bax/Bcl-2 ratio and consequently, increased Cyt C dissociation and caspase 3 activation after caffeine exposure in GBM cell lines (C6 and U87MG). Caffeine at 1 mM reduced the cell viability of both cell lines to <70%. Therefore, to avoid any effects on cell viability, the maximal non-cytotoxic concentration of caffeine on both GBM cells was 0.5 mM (16, 17) (Figure 3A).

Maiese et al. (48) have demonstrated that caffeine also acts increasing the expression of *FOXO1*, important for cell survival and apoptosis regulation. However, the apoptosis mechanisms induced by *FOXO1* need to be elucidated (49, 50). One study has suggested that high *FOXO1* expression after caffeine treatment caused an increased proapoptotic Bim protein expression and, consequently, contributed for the apoptotic effect in U251 GBM cell line (21) (Figure 3A).

#### Effects of Caffeine in Tumor Cell Invasion and Migration

The tumor cell invasion is due to the actin polymerization and the intracellular cytoskeletal organization calcium-dependent proteins (51, 52). In GBM cells, the receptor-mediated calcium mechanism is an important factor for properly invasion, motility, and proliferation of tumor cells (53, 54).

Kang et al. (18) treated several GBM cell lines (U178MG, U87MG, and T98G) with caffeine, and the authors have observed inhibition of proliferation and invasion, in a dose-dependent manner (1-5-10 mM). It was noticed, especially in U178MG cells, the inhibition of IP_3_R, an ion channel responsible for release of calcium from intracellular stores. Moreover, IP_3_R_1_ ion channel subtype was lower expressed in GBM cells, whereas IP_3_R_3_ subtype is more expressed. Caffeine seems to compete to IP_3_R ATP binding even at lower concentrations (10-25-50mM), interfering its function (55, 56) (Figure 2B).

The matrix metalloproteinase (MMP) proteins, especially MMP-2 and MMP-9, are related to cell invasion and angiogenesis (21, 57). Both are regulated by several factors, such as rho-associated protein kinase (ROCK) and ERK (58, 59). Thus, a study has tested caffeine in U87MG GBM cell line and *in vivo* and phosphorylated p38 (p-p38) and MMP-2 were observed as lower expressed. In contrast, the tissue inhibitor metalloproteinase-1 (TIMP-1) presented higher expression (20), which is an important inhibitor of MMP, and plays a crucial role in brain tumor invasion (60). Furthermore, cells exposed to caffeine presented lower levels of cathepsin B, which is responsible for the MMP activation, due to the integrins β1 and β3 inhibition (20) (Figure 2B). The results may explain the lower invasion and adhesion capacity of GBM cells after caffeine exposure (20). The effects of caffeine on GBM cells immigration and evasion are summarized in Figure 2B.

### Compounds Derived From Caffeic Acid

PT93, a compound derived from caffeic acid (CA), has been evaluated regarding its effect on invasion and migration on GBM cell lines (T98G and U251) (22). The authors observed MMP-2 and MMP-9 inhibition in a dose-dependent way (3 and 10μM) (Figure 2B). PT93, a selective MMP inhibitor, seems to present fewer side effects when compared to the first generation of MMP inhibitors *in vitro* studies (61–63), as Batimastat (BB-94) and Marimastat (BB-2516) (64).

In another study, an U87MG xenotransplant mouse has treated with two other CA derivatives, FLVM and FLVZ by targeting the normally overexpressed IL17A, HIF-1α and vascular endothelial growth factor (VEGF). The reduction of these cytokines leads to inhibition of U97MG GBM cell line, mainly because the angiogenesis is also inhibited. Interestingly, the inhibition of all described factors also provides nutrient partition, leading to a lower adipocyte storage due to the glucose, triglycerides, and fat oxidation metabolism reducing (23). In addition, FLVM and FLVZ provide a reduction in the glucose and adipocyte metabolism in the CNS, inhibiting GBM development (23) due to the adipocytes and blood vessel inhibition (65, 66).

### DPG, a New Promising Compound on GBM

Licorice root (*Glycyrhiza glabra*) is widely used in traditional Chinese medicine for its pharmacological and physiological action as antiallergic, antibiotic, antiinflammatory, and antitumor effects (67, 68) (Figure 1B). Glycyrrhizic acid (GA) (C_42_H_62_O_16_), a compound isolated from licorice root, has presented antiinflammatory and antitumor effects on several tumor cell lines such as human hepatoma (HLE), promyelocytic leukemia (HL-60), stomach cancer (KATO III), and prostate cancer (LNCaP e DU-145) by both DNA fragmentation and deregulating genes required for oxidative stress control (69–71). However, toxicity has been observed in *in vivo* models (72).

More recently, one study has exposure U251 GBM cell line to different concentrations of GA (1, 2, and 4 mM), and the authors have observed inhibition on cell proliferation and colony formation, apoptosis stimulation, and significantly decreasing in p65 protein, responsible for nuclear factor kappa B (NF-κB) pathway activating (24).

The NF-κB pathway is constantly activated in GBM, being responsible for the aggressiveness of the disease and regulation of the expression of antiapoptotic genes and cell adhesion and invasion factors (73). Thus, some studies have suggested inhibition of NF-κB pathway could decrease the resistance of tumor cells to chemotherapy and contribute to increase the survival of patients with GBM (74–77).

Following that idea, a recent study has evaluated the DPG (C_42_H_60_K_2_O_16_), a dipotassium salt of GA (Figure 1B), effects in GBM cell lines (25). The authors have demonstrated antitumoral effect in the GBM cell lines, U87MG and T98G, through a decrease of proliferation and an increase of apoptosis. Moreover, after both DPG and TMZ exposure, higher suppressed cell viability was observed in a dose-dependent way. Thus, even low TMZ concentration with half-maximal inhibitory concentration (IC50) of DPG was able to induce U87MG and T98G cell viability reduction (25, 50, 75, 100, 125, 150, 175, and 200 μM) for 6, 12, 18, and 24 h. Thus, a combinatorial therapy, DPG in combination with TMZ, showed a synergistic effect in U87MG and T98G cell lines (25). Interestingly, DPG was able to induce cell viability reduction even in T98G cell line, which presents both the hypermethylated *MGMT* promoter and mutated *P53* gene, making the phenotype more aggressive and resistant to the action of TMZ than the ones with wild-type *P53* (78), since p53 is fundamental in regulating the cell cycle arrest and the entry in the apoptotic process (79, 80).

In addition, DPG (18 mM and 24 mM for U87MG and T98G cells, respectively) antitumoral effect was related to NF-κB pathway suppression by *IRAK2* and *TRAF6*-mediating miR-16 and miR-146a, respectively (25) (Figure 3B), and consequently increasing the TMZ-induced apoptosis. Finally, the authors have also showed that DPG was able to inhibit the subpopulation of stem cells essential for tumor formation, survival, and recurrence (25). Further *in vivo* studies may elucidate the antitumor effect of DPG as an alternative treatment for GBM. The effects of GA and DPG on NF-κB pathway are presented in Figure 3B.

More recently, it was demonstrated that the cytotoxic effect of DPG was time- and dose-dependent also in U251 and U138MG and DPG (IC50: 32 mM and 20 mM for 48 h, respectively) inhibited cell viability by activating apoptosis, inhibiting cell proliferation and stem cell subpopulation formation through miR-4443 and miR-3620 upregulation. Both miRs are responsible for post-transcriptional inhibition of the NF-κB pathway by *CD209* and *TNC* modulation on U251 and U138MG cell lines. The authors have concluded that DPG presents an antimigratory effect on GBM cell lines by inhibition of cancer stem-like cells, evidenced by inhibition of neurosphere formation (unpublished data).

### Curcumin, a Turmeric Compound With Antitumor Effect on GBM

Curcumin (diferuloylmethane; C_21_H_20_O_6_) (Figure 1C) is the main compound found in turmeric, an Indian spice derived from *Curcuma longa Linn* (81). It has been demonstrated that the curcumin has present antioxidant and antiinflammatory effects (81), mainly in diabetes (82), Alzheimer's disease (83), and hepatitis (84).

Curcumin seems to play a role in a variety of pathways as proinflammatory cytokines (TNF-alpha, IL1, IL6, IL8) inhibition, wingless-related integration site (WNT) suppressing, mitogen-activated protein kinase pathway (MAPK) and Janus kinase/signal (JAK/STAT) activation (85–88). Furthermore, curcumin is highly lipophilic, which makes it permeable to the BBB (89, 90), making it further viable *in vivo* therapy.

A recent study has evaluated the curcumin effect over miRs in the GBM cell line, A172. Interestingly, the authors have observed that curcumin was able to downregulate the serine or threonine kinase pathway (AKT) and p53-BCL2 pathways by overexpressing several miRs such as miR-223-3p, miR-133a-3p, miR-181a-5p, miR-34a-5p, miR-30c-5p, and miR-1290 and also downregulating the expression of miR-27a-5p, miR-221-3p, miR-21-5p, miR-125b-5p, and miR-151-3p (31) (Figure 3B). AKT and p53-BCL2 pathways are related to proliferation and cellular growth, metabolism, apoptosis, and autophagy (31). In contrast, the authors also observed that in GBM curcumin-treated cells, the miR-27a expression level was reduced and its target gene, *CHOP*, was overexpressed leading the cells to paraptosis, mainly due to endoplasmatic reticulum (ER) instability (31) (Figure 3B).

Other studies have also observed that curcumin enhanced the expression of miR-326 (28), miR-378 (27), and miR-21 (29) in GBM cell lines. Thus, Yin et al. (28) have observed that U87MG and U251 cells presented a marked increase of curcumin-induced cytotoxicity and apoptosis and a decrease of proliferation and migration in GBM cells. Moreover, the authors have found that combination treatment of miR-326 mimics and curcumin caused significant inhibition of the SHH/GLI1 pathway in cells compared with either treatment alone, independent of p53 status. Furthermore, *in vivo*, the curcumin-induced miR-326 expression reduces tumor volume and prolonging the survival period compared with either treatment alone. The results support an important role of miR-326 in enhancing the chemosensitivity of glioma cells to curcumin. Similar results were observed in another study, in which the authors have evaluated the curcumin effect on U87MG cells stably expression miR-378. Cells were unable to form colonies compared with control cells, indicating a lower survival rate when treatment of curcumin and miR-378 was combined. In conclusion, curcumin can inhibit MAPK pathway activation by overexpressing miR-378 (27).

Tan et al. (29) have transfected deoxycholic acid-conjugated polyethylenimine (DP) micelles containing curcumin and miR-21 antisense oligonucleotide (ASO) into C6 GBM cell line, aiming knockdown the miR and enhance the expression of proapoptotic target genes. The authors have significantly observed cell viability decreases and apoptosis stimulation when compared to control cells (DP-curcumin). The anticancer effect of DP-curcumin-mir-21ASO complex was also evaluated in *in vivo* essays and it was noticed a significantly tumor growth decrease compared with miR-21ASO and curcumin alone in animals. Curcumin seems increasing miR-21 and consequently inhibiting *PDCD4* and *PTEN* target genes, resulting in cell death.

Glioma stem cells (GSCs) were transfected with lentivirus-GFP-miR-145, upregulating miR-145 and treated with demethoxycurcumin (DC), a curcumin compound. It was observed a greater inhibition in tumor growth and stimulation of apoptosis in both *in vivo* and *in vitro* when compared to control and to monotherapy (30). Combined lentivirus-GFP-miR-145 plus DC was able to inhibit miR-145 target gene *SOX2*, leading to downstream beta-catenin downregulation, responsible for transcriptional activation of *CCND1* and *C-MYC* (30).

Interestingly, curcumin appears to suppress AP-1 and NF-kB pathways leading to chemosensitization (91). Therefore, Wu et al. (26) have treated U87MG cells with curcumin and TMZ alone, and a higher expression of miR-146a was observed in curcumin-treated cells, in a dose-dependent way. The enhancement of miR-146a leads to inhibition of p65 and phosphorylation IκBα and consequently, suppressing NF-kB, increasing the TMZ-induced apoptosis (26).

The effects of curcumin on several pathways at GBM cell lines are summarized in Figures 3A,B. In addition, this study indicated that only DPG and curcumin have an antitumoral effect on GBM cell lines through miR modulation (Table 2, Figure 3).

Furthermore, curcumin in combination with radiation has presented a synergistic effect in U87MG and T98G GBM cell lines, using different concentrations (ranging from 5 to 25 μM and 3.25–26 μM in U87 and T98 cells, respectively) and dosages (2 Gy or 4 Gy of irradiation). In U87 cells, curcumin and radiation exerted synergism in most of the tested combinations, and the highest synergy was monitored when curcumin was given at its IC50 (10 μM). In T98 cells, the highest levels of synergy were observed at higher curcumin concentrations, particularly at 26 μM, possibly due to the resistance of those cells to both chemotherapy and radiotherapy. The combinatorial treatment arrested both cell lines at the G2/M phase to a higher extent than radiation or curcumin treatment alone. In addition, it was also observed a synergistic effect of curcumin when combined with TMZ resulting in increased tumor cell death (92).

In accordance, Yin et al. (93) have treated U87MG cell line with curcumin in combination with TMZ. The authors have observed that apoptosis was enhanced, *in vitro* and *in vivo*, in the GBM cells by both generation of reactive oxygen species production and phosphorylated AKT and mTOR suppression. These data indicated that blockage of AKT/mTOR signaling appeared to contribute to the elevated apoptosis caused by the combination treatment of curcumin and TMZ. Further, in the U87MG xenograft mouse model, the combination treatment with curcumin and TMZ showed a significantly enhanced inhibition of tumor growth compared with single treatments. A similar trend was observed by the authors in the measurement of tumor weight.

### An Overview of Euphol Antitumor Effect

In traditional medicine, the base extracts of species of the genus *Euphorbia* (*Euphorbiaceae*) are often used as a form of treatment for ulcers and warts (32, 94–96). The euphol (C_30_H_50_O), a tetracyclic triterpene alcohol, is the main constituent of the *Euphorbia tirucalli* known as aveloz (Figure 1D) and it has been observed to have antiinflammatory effects as antiviral, analgesic, and nociceptive properties. Recently, a potential antitumor activity has been also noticed by euphol (97–99). Thus, euphol was able to decrease cell viability in CS12 gastric cancer cell line (100, 101).

Silva et al. (32) have evaluated the euphol antitumor effect in 12 human gliomas and GBM cell lines comprising seven adults (U87MG, U373, U251, GAMG, SW1783, SNB19), five pediatric glioma cell lines (RES186, RES259, KNS42, UW479, and SF188), two primary cultures (HCB2 and HCB149), and one normal astrocyte cell line (NHA) for cytotoxic assays. The pediatric cell lines have showed more euphol sensibility than adult and primary cultures. Moreover, euphol had a higher selective cytotoxicity index (0.64-3.36) than TMZ (0.11-1.13). When combined, euphol and TMZ treatments seem to have a synergistic effect [combination index (CI <1) in 67% (8/12) of the glioma cell lines investigated (mean CI values: range: 0.48–0.96)]. However, no effect was found on cell cycle distribution, invasion, and colony cell formation (90). In addition, the authors have compared both drug-sensitive (GAMG) and drug-resistant (U373) cell lines using euphol dose at 15 μM, which was able to inhibit GAMG and U373 proliferation by 35.44 and 28.71%, respectively. Moreover, at 15 μM euphol suppressed cell viability of GAMG cells by 88.86% and U373 cells by 13.9%. These data suggest that euphol seems to have predominantly cytotoxic effects on the anchorage-dependent growth of both malignant glioma cell lines (32). Finally, euphol also exhibited antitumoral and antiangiogenic activity *in vivo*, using the chicken chorioallantoic membrane assay, with synergistic TMZ interactions in most above GBM cell lines (32). In conclusion, euphol exerted *in vitro* and *in vivo* cytotoxicity against glioma cells, through several cancer pathways. These findings provide experimental support for further development of euphol as a novel therapeutic agent for GBM.

In addition to euphol, the genus *Euphorbia* also has diterpenes as important bioactive constituents some already approved for precancerous conditions (33, 102–104). One diterpene that was approved for human use for the treatment of actinic keratosis, ingenol-3-angelate (I3A) (Picato®), from *Euphorbia peplus* demonstrated great antineoplastic potential evaluated in clinical trials for the effective treatment of basal cell carcinoma and squamous cell carcinoma through the modulation of PKC signaling (105–109). Some studies have also revealed diterpenes as promising modulators of multidrug resistance (MDR) in tumor cells and also showing *in vivo* antiinflammatory activity (110).

Recently, the cytotoxic potential of a new esters of semisynthetic ingenol from *E. tirucalli*, the derivative ingenol-3-dodecanoate (Ingenol C-IngC) was reported. IngC showed higher efficacy when compared to I3A and ingenol 3,20-dibenzoate (IDB) from E. esula L on esophageal cancer cell lines, two important ingenol diterpenes that can promote PKC activation and anticancer activity (33, 108, 111). In a panel of 11 glioma cell lines, IngC acted as a potent inhibitor of protein kinase C (PKC) activity by PDK1 inhibiting and consequently tumor invasion and migration impairment through Wnt/β-catenin (β-C) pathways by lower concentration the effector protein β-C (93) (Figure 2B). The different models of glioma cell lines exhibited a heterogeneous profile of response to IngC. At a fixed dose of 10 μM, 9.1% (1/11) of cell lines were resistant, 36.4% (4/11) were moderately sensitive, whereas 54.5% (6/11) were classified as highly sensitive. These findings identify IngC as a promising lead compound for the development of new cancer therapy and they may guide the search for additional PKC inhibitors.

Despite euphol's antitumor action, some reports have demonstrated that exposure to crude *Euphorbia tirucalli* may be a risk factor for Burkitt's lymphoma, as a result its performance as a genotoxic agent (97, 112) indicating that further studies are needed to define the potential therapeutic use of euphol. The effects of IngC and euphol on different GBM cell pathways are summarized in Figures 2B, 3A, respectively.

## Author Contributions

GB, MB, AS, and MO: wrote the manuscript. GB, MB, AS, JZ, and TR: performed the research. MB and MO: designed the figures. MO: designed the research. All authors approved the submitted manuscript.

## Funding

The financial support provided by the National Council for Scientific and Technological Development (CNPq) scholarship #149884/2019-2, #137689/2020-9, and #122513/2021-5 is gratefully acknowledged.

## Conflict of Interest

JZ is employed by Verdi Cosmetics LLC, Joanópolis, São Paulo, Brazil. The remaining authors declare that the research was conducted in the absence of any commercial or financial relationships that could be construed as a potential conflict of interest.

## Publisher's Note

All claims expressed in this article are solely those of the authors and do not necessarily represent those of their affiliated organizations, or those of the publisher, the editors and the reviewers. Any product that may be evaluated in this article, or claim that may be made by its manufacturer, is not guaranteed or endorsed by the publisher.
